# A Safe and Portable CMUT Array Ultrasonic System for Bubble Sizing in Industrial Silicone Sealing Rings

**DOI:** 10.3390/mi17080882

**Published:** 2026-07-24

**Authors:** Changde He, Shuo Liu, Hanchi Chai, Dandan Li, Chengrui Liu, Wanjia Gao, Yuhua Yang, Licheng Jia, Guojun Zhang, Renxin Wang, Jiangong Cui, Wendong Zhang

**Affiliations:** State Key Laboratory of Extreme Environment Optoelectronic Dynamic Measurement Technology and Instrument, North University of China, Taiyuan 030051, China

**Keywords:** ultrasonic measurement, CMUT array, silicone bubble detection, time-of-flight (TOF), ultrasonic velocity

## Abstract

To address the need for bubble detection in industrial silicone sealing rings, this paper presents a compact non-invasive ultrasonic monitoring system based on a capacitive micromachined ultrasonic transducer (CMUT) array, aiming to overcome the limitations of conventional X-ray inspection in terms of safety, portability, and real-time in situ monitoring. The system comprises two 8.8 mm × 8.8 mm CMUT arrays with associated transmitting and receiving circuitry. The silicone thickness is determined using the time-of-flight (TOF) method, while bubble size is quantitatively estimated by combining received signal amplitude analysis, which characterizes bubble-induced attenuation, with correlation function evaluation. Experimental measurements on industrial-grade silicone samples and finite element simulations demonstrate that the system achieves a spatial resolution of 0.5 mm and effectively captures attenuation variations caused by bubbles. The integrated strategy of TOF, amplitude analysis, and correlation assessment ensures reliable non-destructive evaluation. Compared with X-ray inspection, the proposed system is safer, more portable, and suitable for real-time on-site monitoring, thereby significantly improving quality control efficiency in silicone manufacturing. This study provides a novel CMUT-array-based solution for quantitative bubble detection in silicone media, offering both high resolution and practical application potential.

## 1. Introduction

Silicone sealing rings are widely used in industrial pipeline systems due to their excellent elasticity, chemical stability, and temperature resistance. Their ability to undergo elastic deformation enables them to effectively compensate for surface irregularities and maintain reliable sealing performance under various operating conditions [1]. In addition, silicone materials exhibit excellent environmental adaptability and long-term stability, making them suitable for applications requiring high reliability and durability [2,3]. However, defects generated during material preparation and manufacturing processes, especially internal bubbles, may significantly degrade the structural integrity and sealing performance of silicone components [4]. Therefore, accurate and non-destructive detection of internal bubbles is essential for improving the reliability and quality control of silicone sealing products.

Various non-destructive testing methods have been investigated for detecting internal defects in polymer materials. Currently, X-ray and Lambda-ray penetration testing are commonly used for silicone bubble inspection because defects can be identified based on differences in radiation attenuation caused by density variations within materials. However, these methods suffer from several limitations, including potential radiation hazards to operators, strict requirements for protective facilities, high equipment costs, and limited suitability for real-time industrial inspection [5,6]. X-ray computed tomography (CT) technology can provide a three-dimensional visualization of internal structures, but its complex operation, high cost, and relatively low inspection efficiency restrict its application in large-scale production environments.

Ultrasonic testing has attracted increasing attention as an alternative technique for internal defect detection due to its advantages of safety, low cost, high penetration capability, and compatibility with real-time monitoring [7,8,9,10]. Conventional ultrasonic inspection methods, such as pulse-echo and transmission measurements, have been widely applied in defect detection of various materials. However, for thin polymer components such as silicone sealing rings, acoustic coupling effects, near-surface interference, and complex scattering mechanisms caused by small defects may affect detection accuracy. Furthermore, conventional ultrasonic systems often require bulky transducers and mechanical scanning structures, which limits their integration into compact and automated inspection platforms [11].

With the development of micro-electromechanical system (MEMS) technology, micromachined ultrasonic transducers have attracted increasing attention for compact ultrasonic sensing applications. According to their operating principles, MEMS ultrasonic transducers can mainly be categorized into capacitive micromachined ultrasonic transducers (CMUTs) [12] and piezoelectric micromachined ultrasonic transducers (PMUTs) [13]. Compared with PMUTs, CMUTs provide advantages including higher electromechanical coupling efficiency, broader operating bandwidth, excellent sensitivity, and potential for array integration [14,15,16,17,18,19,20,21]. These characteristics make CMUTs promising candidates for miniaturized and high-performance ultrasonic inspection systems. However, most existing CMUT-based studies have focused on biomedical imaging, acoustic sensing, and fluid-related applications, while quantitative evaluation of bubble defects in industrial silicone components using CMUT arrays remains insufficiently explored. The acoustic performance of the fabricated CMUT arrays, including center frequency, bandwidth, and sensitivity, is experimentally characterized and discussed in this work.

**Figure 1:**
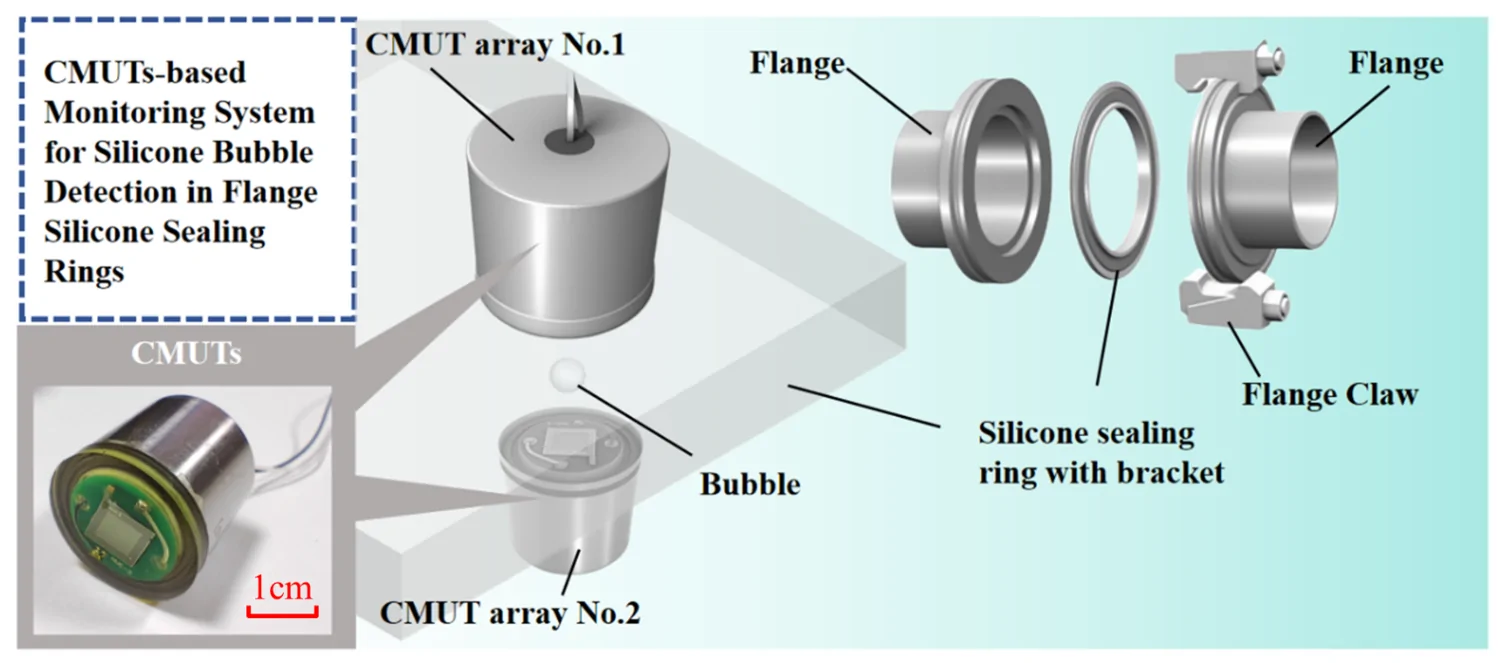
The silicone bubble-monitoring system designed based on CMUT arrays.

In this work, a compact non-invasive silicone bubble monitoring system based on dual CMUT arrays is proposed. As shown in Figure 1, the proposed system separates silicone thickness measurement and bubble detection functions using two CMUT arrays. The first CMUT array performs pulse-echo measurement to determine silicone thickness through time-of-flight (TOF) analysis, while the second CMUT array receives transmitted acoustic signals and evaluates bubble-induced attenuation for quantitative bubble sizing. Furthermore, a correlation model between bubble size, silicone thickness, and received voltage variation is established to estimate bubble dimensions. The proposed CMUT-based system provides a compact, low-cost, and radiation-free solution for silicone quality inspection, demonstrating significant potential for real-time industrial applications.

## 2. Theory

**Figure 2:**
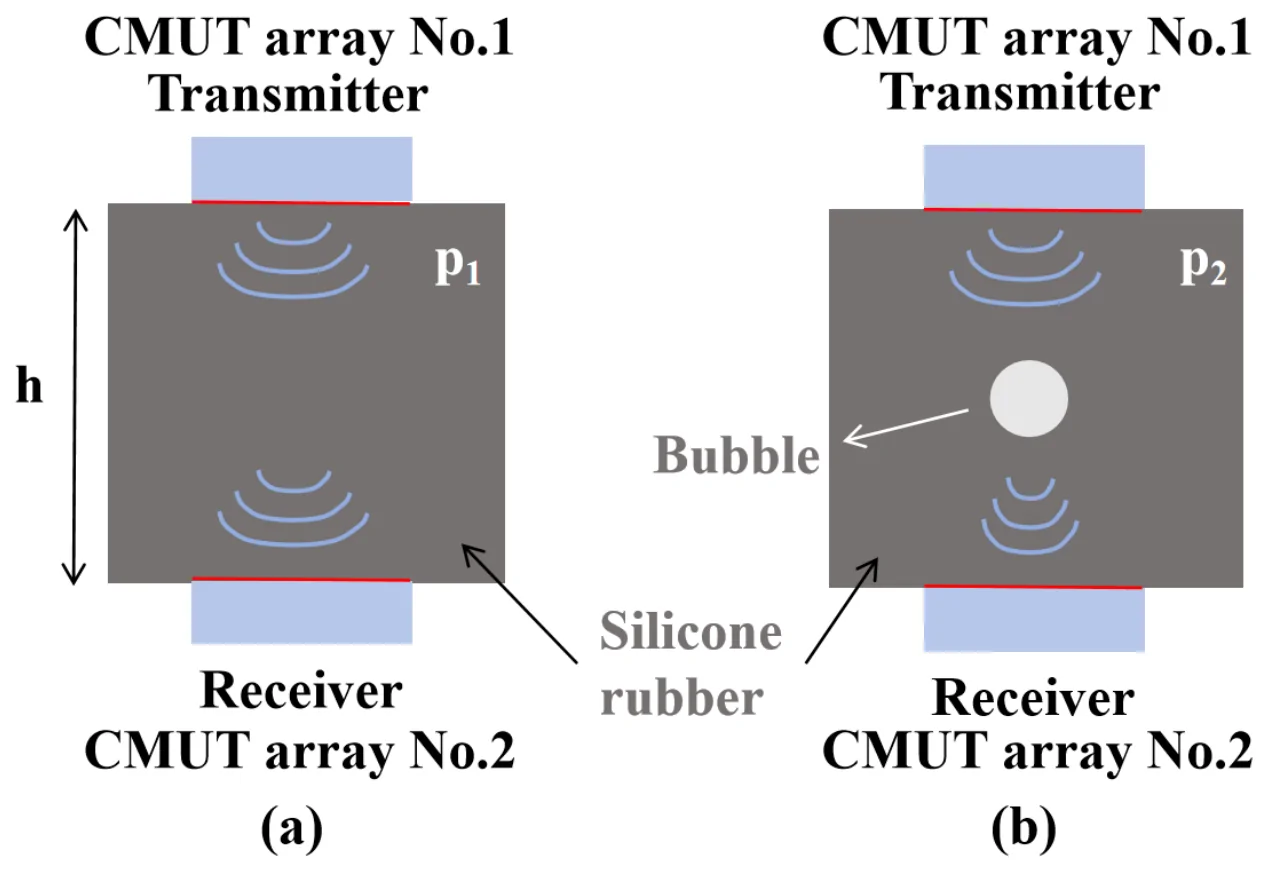
Schematic diagram of ultrasonic propagation in silicone. (**a**) Schematic diagram of ultrasonic propagation without bubbles. (**b**) Schematic diagram of ultrasonic propagation in the presence of bubbles.

Due to the presence of coupling signals during acoustic testing, echo signals overlap with coupling signals in near-surface echo detection, making it difficult to obtain bubble information via echo imaging methods [22]. Consequently, we adopt the principle that bubbles in silicone cause attenuation of propagating acoustic signals. The schematic diagram of bubble detection in silicone is illustrated in Figure 2, where two CMUT arrays are placed on opposite sides of the silicone component. The transmitting CMUT array emits pulse signals and receives reflected echo signals, which are used to determine the silicone thickness through time-of-flight (TOF) measurement. Meanwhile, the opposite CMUT array receives transmitted acoustic signals after propagation through the silicone component. As shown in Figure 2a, p1 represents the received acoustic pressure signal when no bubbles exist in the silicone, while Figure 2b shows p2, representing the received acoustic pressure signal when bubbles are present. In comparison, when bubbles are present in the silicone, the voltage received by the CMUT array on the opposite side of the transmitting end decreases. This attenuation in the received voltage is utilized to detect bubbles in the silicone.

**Figure 3:**
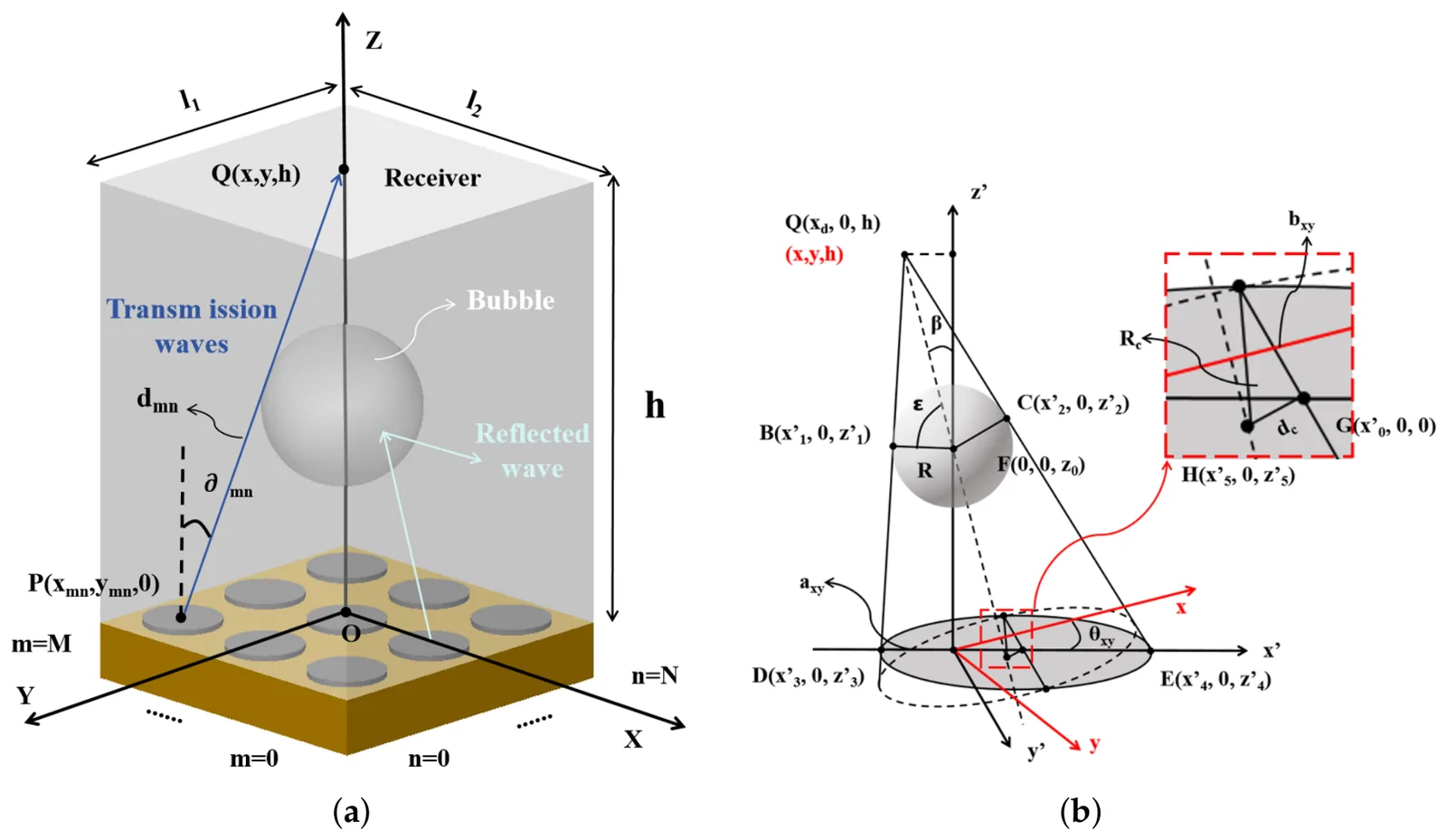
Working principle of detecting bubbles in silicone gel. (**a**) Schematic diagram of sound pressure analysis. (**b**) Geometric schematic diagram for analyzing the projected area of bubbles.

To establish an accurate relationship between bubbles in silicone and the received acoustic pressure, we derived theoretical expressions for the variation in received voltage under different bubble sizes based on acoustic theory [23]. Figure 3a presents a three-dimensional schematic of acoustic signal propagation in the array of the sensitive cell within the CMUT element. The radius of a single sensitive cell in the designed CMUT array is smaller than one wavelength; thus, each sensitive cell in the CMUT array can be regarded as a circular piston with the same emitting surface area s, thereby simplifying the vibration analysis model of the cell [24]. The acoustic pressure emitted by each sensitive cell was calculated based on acoustic propagation theory. Neglecting thermal losses of acoustic energy, the expression for the acoustic pressure at point Q(x,y,h) in the absence of bubbles in silicone is given as [25]

(1) $$p_{Q1}\left(x,y,h\right)=\sum_{m=1}^{M}\sum_{n=1}^{N}\frac{jT_{p1}T_{p2}\rho_{z}\omega s}{2\pi d_{mn}}\nu_{mn}e^{\left(-jkd_{mn}\right)}\cdot D\left(\partial_{mn}\right)$$

In the equation, j is the imaginary unit; s denotes the density of silicone; $V_{in}$ is the angular frequency of the acoustic wave; k represents the wave number; (m, n) is the index of the CMUT array sensitive cell located in the m-th row and n-th column; $V_{mn}$ denote the vibration velocity of the CMUT array sensitive cell; and $d_{mn}$ and $\partial_{mn}$ denote the length of PQ and the angle between PQ and the z-axis, respectively. The values of $d_{mn}$ and $\partial_{mn}$ can be given by the following formulas.

(2) $$\;d_{mn}=\sqrt{{\left(x-x_{mn}\right)}^{2}+{\left(y-y_{mn}\right)}^{2}+h^{2}}$$

(3) $$\partial_{mn}=\tan^{-1}\frac{h}{\sqrt{{\left(y-y_{mn}\right)}^{2}+{\left(x-x_{mn}\right)}^{2}}}$$

In both equations, h represents the distance between the two CMUT arrays. D($\partial_{mn}$) is the directivity function of the CMUT array sensitive cell at point P, which is expressed as

(4) $$D\left(\partial_{mn}\right)=\frac{2J_{1}\left(ka\sin\partial_{mn}\right)}{ka\sin\partial_{mn}}$$

where Ra denotes the radius of the sensitive cell, and $J_{1}$ (kasin$\partial_{mn}$) represents the first-order Bessel function of the first kind.

$T_{p1}$ and $T_{p2}$ denote the acoustic transmission coefficients from the polyurethane matching layer to the tested silicone, and from the tested silicone to the polyurethane matching layer, respectively. These coefficients can be given by the following formulas.

(5) $$T_{p1}=\frac{4Z_{1}Z_{2}}{{\left(Z_{1}+Z_{2}\right)}^{2}}$$

(6) $$T_{p2}=\frac{4Z_{2}Z_{3}}{{\left(Z_{2}+Z_{3}\right)}^{2}}$$

where $Z_{1}$ and $Z_{3}$ denote the acoustic impedances of the polyurethane matching layer, while $Z_{2}$ represents the acoustic impedance of the tested silicone. The acoustic impedance can be obtained from the formula.

(7) Z=ρc

where ρ is the material density and c is the speed of sound in the material.

Figure 3b presents a geometric schematic diagram of the bubble projection area when bubbles exist in silicone. Due to the significant difference in acoustic impedance between silicone and gas, the emitted acoustic waves undergo reflection at the silicone–bubble interface, such that the acoustic signals capable of passing through the bubble are negligible. In this case, the acoustic energy emitted by the sensitive cells of CMUT array No.1 within the gray bubble projection area in the figure cannot be transmitted to CMUT array No.2. It is assumed that the bubble center lies on the axis of CMUT array No.1. Based on the geometric principles illustrated in the figure, the projection area can be calculated as follows:

(8) $$\frac{{\left(x^{\prime }-x_{0}\right)}^{2}}{a_{x,y}^{2}}+\frac{y^{\prime 2}}{b_{x,y}^{2}}=1$$

(9) $$\left[x^{\prime },y^{\prime }\right]=\left[x,y\right]\left[\begin{array}{cc} \cos\theta_{x,y} & -\sin\theta_{x,y}\\ \sin\theta_{x,y} & \cos\theta_{x,y} \end{array}\right]$$

Accordingly, the acoustic pressure at point Q (x, y, h) in the presence of bubbles is given by

(10) $$p_{Q2}\left(x,y,h\right)=\sum_{m=1}^{M}\sum_{n=1}^{N}\frac{jT_{p1}T_{p2}\rho_{s}\omega s}{2\pi d_{mn}}v_{mn}e^{\left(-jkd_{mn}\right)}\cdot D\left(\partial_{mn}\right)$$

(11) $$\left(\frac{{\left(x_{mn}^{\prime }-x_{0}\right)}^{2}}{a_{x,y}^{2}}+\frac{y_{mn}^{\prime 2}}{b_{x,y}^{2}}\geq 1\right)$$

(12) $$\left[x_{mn}^{\prime },y_{mn}^{\prime }\right]=\left[x_{mn},y_{mn}\right]\left[\begin{array}{cc} \cos\theta_{x,y} & -\sin\theta_{x,y}\\ \sin\theta_{x,y} & \cos\theta_{x,y} \end{array}\right]$$

Finally, by combining the received acoustic pressure with the receiving sensitivity $S_{C}$ of the CMUT array, the received voltages of CMUT array No.2 in the absence and presence of bubbles in silicone can be obtained, respectively.

(13) $$V=\frac{S_{c}}{l_{1}l_{2}}{\;\int \int \;}_{D}p_{Q}\left(x,y,h\right)dx\;dy$$

(14) $$\left(D:-l_{1}/2\leq x\leq l_{1}/2,-l_{2}/2\leq y\leq l_{2}/2\right)$$

The $l_{1}$ and $l_{2}$ are the two side lengths of CMUT array No.2, respectively.

To simplify the calculation and eliminate the influence of system parameter fluctuations and environmental parameters, the concept of normalization is adopted, and the relative voltage change $\alpha_{\Delta V}$ is used as the judgment signal for bubble size recognition, which can be expressed as

(15) $$\alpha_{\Delta V}=\frac{V_{1}-V_{2}}{V_{1}}$$

where $V_{1}$ represents the received voltage of CMUT array No.2 when there is no bubble in the silicone, and $V_{2}$ denotes the received voltage of CMUT array No.2 when bubbles are present in the silicone. From the aforementioned equations, it can be derived that the received voltage is related to the bubble size and the thickness of the silicone.

Testing and calculating the thickness of silicone requires the use of ultrasonic time-of-flight (TOF). By measuring the TOF, the propagation speed c of ultrasonic waves in various media can be calculated as follows:

(16) c=d/t

In the equation, d denotes the ultrasonic propagation distance and t represents the ultrasonic propagation time. Once the propagation speed of silicone is calibrated, the thickness of the silicone can be derived based on the measured ultrasonic TOF.

Thus, by establishing the relationship between silicone thickness, bubble size, and relative voltage variation, the size of bubbles present in silicone can be detected in real-time.

## 3. Design and Manufacturing

**Figure 4:**
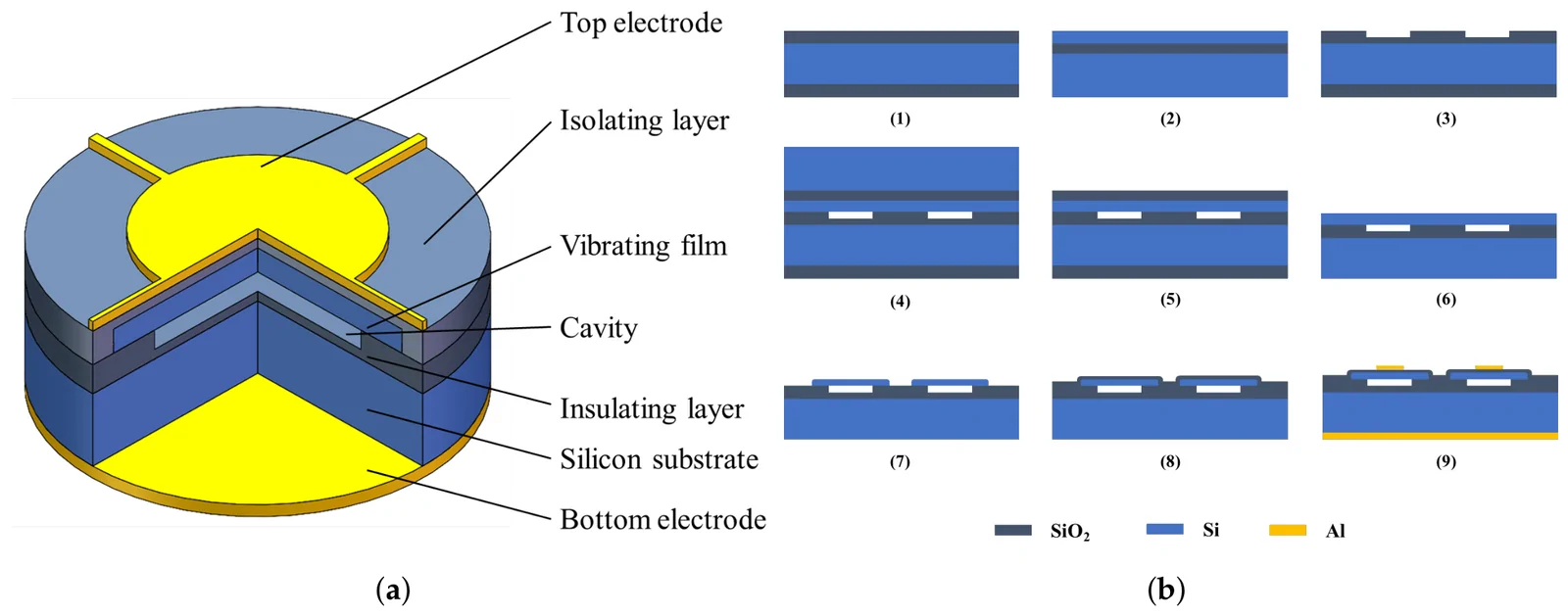
The structure and processing flow of the CMUT array we produced. (**a**) Structural diagram of a single sensing cell in the CMUT array. (**b**) Manufacturing process flow diagram of the CMUT array.

As shown in Figure 4a, the CMUT structure comprises, from top to bottom, a top electrode, an isolation layer, a vibrating membrane, an insulating layer, a silicon substrate, and a bottom electrode. CMUT operates based on the capacitive effect and features two working modes. In the transmit mode, applying a specific voltage to the top and bottom electrodes induces vibration of the membrane, thereby generating ultrasonic waves. In the receive mode, ultrasonic signals cause vibration of the sensor membrane; the generated charges then pass through backend circuits such as charge amplifiers, resulting in a voltage signal. The CMUT device is fabricated using silicon fusion wafer-bonding technology, which enables direct bonding of two silicon surfaces at high temperatures, characterized by high bonding strength and excellent hermeticity. The process flow diagram is illustrated in Figure 4b.

This process requires the use of two wafers: one oxidized single-crystal silicon wafer and one silicon-on-insulator (SOI) wafer (step 1 and 2). First, reactive ion etching (RIE) is employed to etch 650 nm of silicon dioxide to form the cavity, while retaining a 150 nm-thick oxide layer as the device’s insulating layer. This layer prevents contact between the membrane and the substrate, ensuring electrical isolation (step 3). Next, the SOI wafer and the oxidized silicon wafer with patterned etched cavities are pre-bonded under vacuum at room temperature. The pre-bonded wafers are then annealed at high temperatures, with a maximum temperature reaching 1150 °C, which constitutes the fusion bonding process (step 4). Subsequently, the handle layer and buried oxide (BOX) layer of the SOI wafer are removed. The membrane of the device layer is released through chemical mechanical polishing (CMP) and wet etching processes (step 5 and 6). Deep Reactive Ion Etching (DRIE) is utilized for front-side silicon etching to form isolation trenches and grooves (step 7). At this stage, an SOI wafer with cavities is fabricated, and a 100 nm-thick silicon oxide layer is deposited on the device layer using Plasma-Enhanced Chemical Vapor Deposition (PECVD) (step 8). Finally, an aluminum metal layer is sputtered and patterned on the device layer side of the cavity SOI to form the top electrode of the CMUT device. Another aluminum metal layer is sputtered on the handle layer side of the cavity SOI without patterning, forming the bottom electrode of the CMUT device (step 9). Annealing is performed to create an ohmic contact between the bottom electrode and the highly doped silicon substrate.

**Figure 5:**
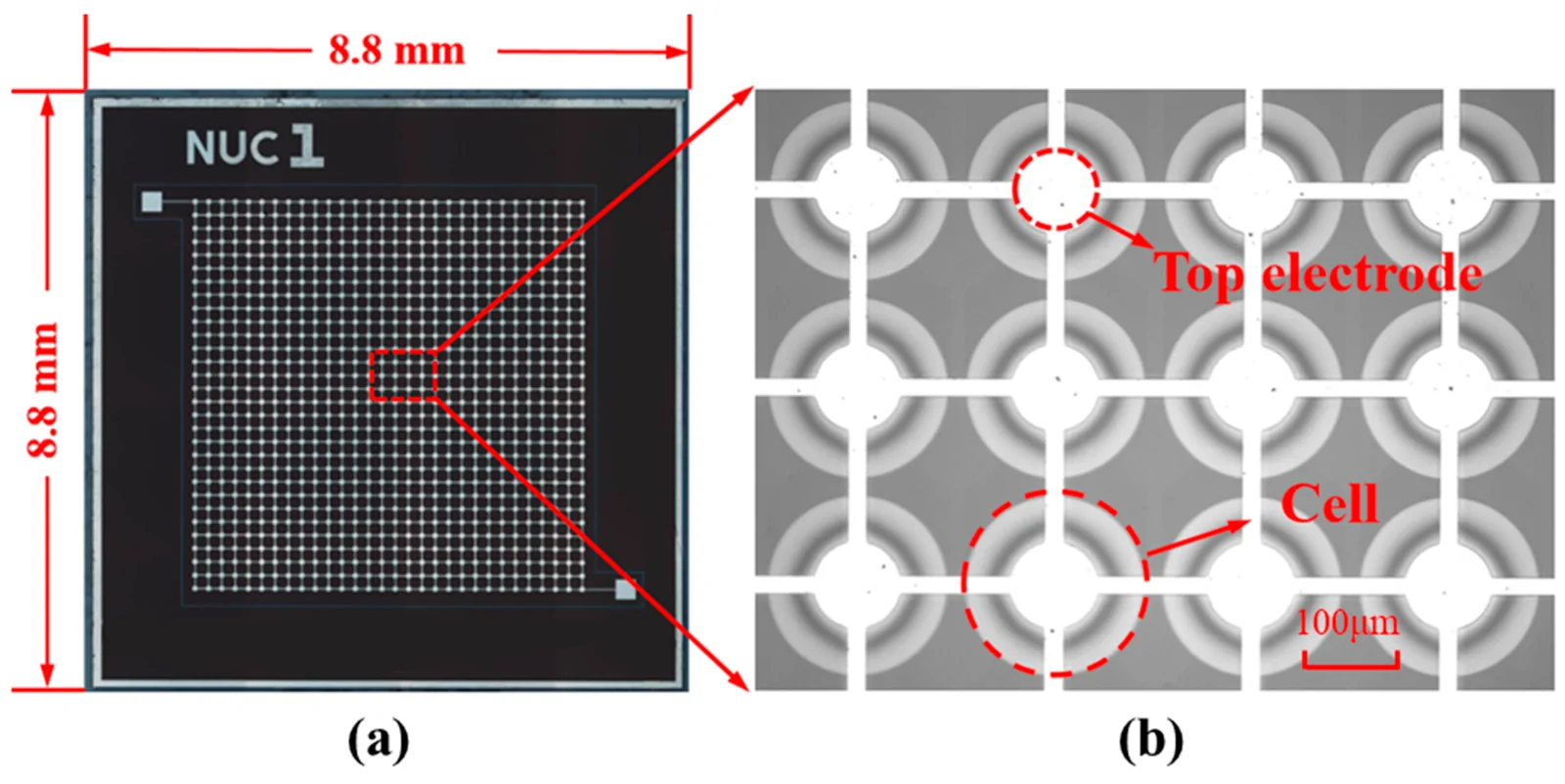
Optical microscope images of the fabricated CMUT array. (**a**) Overview of the CMUT array and close-up view of a sensing cell in the CMUT array. (**b**) Cross sectional electron microscopy (SEM) image and partial enlarged view of the structure of a single sensing unit in CMUT array.

The planar scanning electron microscopy (SEM) image of the CMUT array designed in this work is presented in Figure 5, with a dimensions 8800 µm × 8800 µm. Specifically, Figure 5 show the optical microscopy images of the CMUT array, respectively, after fabrication and a close-up view of a single sensing cell. The detailed parameters of the CMUT array in this design are listed in Table 1.

To evaluate the acoustic performance and confirm the broadband characteristics of the fabricated CMUT arrays, pulse-echo measurements were conducted. The ultrasound pulse transmitter and receiver (DPR300, JSR Ultrasonics, Pittsford, NY, USA) apply short electrical pulse excitation to the transducer, and the resulting acoustic echo signals were acquired and digitized utilizing an oscilloscope (DSOX3024T, Keysight, Santa Rosa, CA, USA). A Fast Fourier Transform (FFT) was subsequently applied to the recorded time-domain echo signal to obtain the frequency response spectrum.

**Figure 6:**
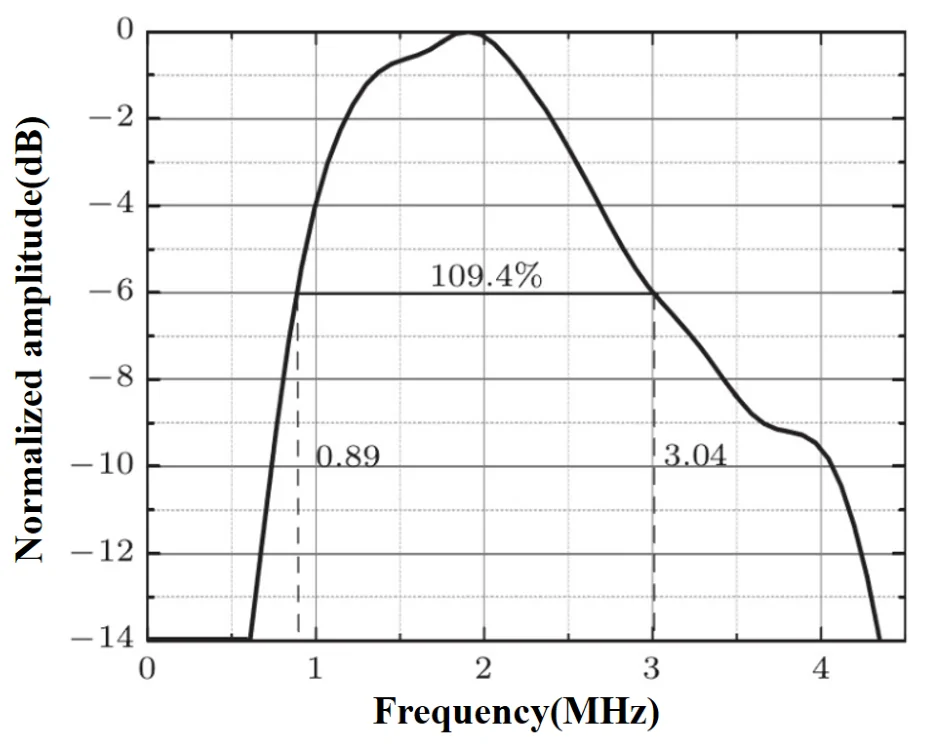
The normalized frequency response spectrum of the CMUT array.

As illustrated in Figure 6, the normalized frequency spectrum demonstrates excellent acoustic bandwidth. The lower and upper −6 dB cut-off frequencies are measured at 0.89 MHz and 3.04 MHz, respectively. This corresponds to a center frequency of 1.965 MHz and a wide fractional bandwidth of 109.4%. Such broadband performance ensures high resolution and rapid transient response, which are crucial for the accurate identification and monitoring of internal bubbles within the silicone sealing rings.

## 4. Simulation Verification

**Figure 7:**
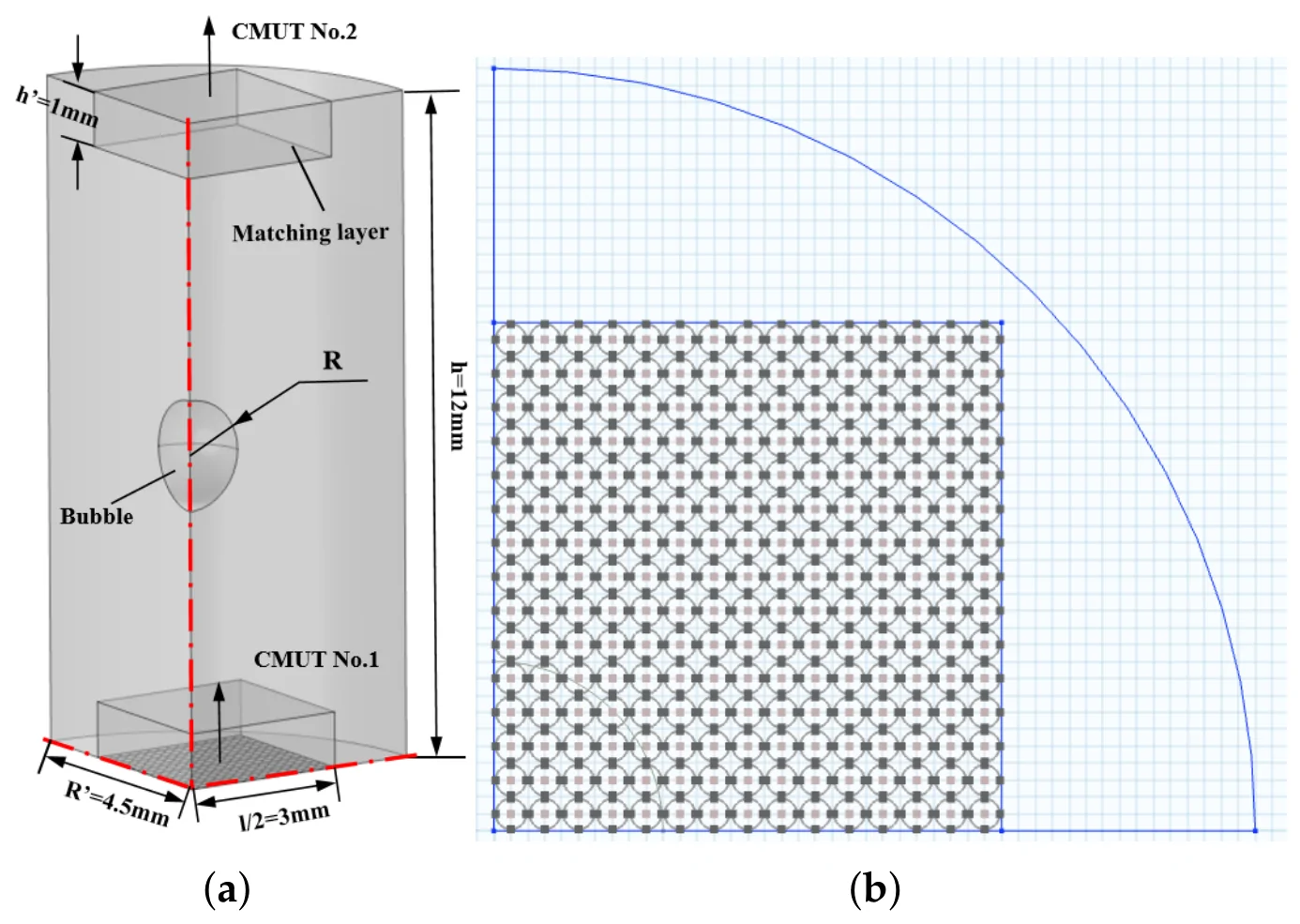
Bubble simulation structure. (**a**) Finite element simulation model. (**b**) Sensing unit array used in the simulation, which is consistent with the designed CMUT array.

To validate the feasibility of the principle for measuring bubbles in silicone, we developed a finite element model corresponding to our designed sensor using COMSOL Multiphysics 6.2 (COMSOL Inc., Stockholm, Sweden). This model simulates the impact of bubbles of varying sizes in silicone on the received voltage during acoustic pressure emission. Considering the structural symmetry of the original device and computational efficiency, a 1/4 simplified model was adopted in the simulation. This simplification leverages the axisymmetric characteristics of the device’s geometry, reducing computational burden while ensuring the accuracy of simulation results through symmetric boundary conditions and avoiding redundant computational costs associated with a full model. Figure 7a depicts the schematic of the finite element model we established. The pressure acoustics module was employed for silicone and the impedance matching layer, which was made of polyurethane. A plane wave radiation boundary condition was applied to the cylindrical surface of the silicone, and symmetry conditions were set on the symmetric planes. Transient acoustic simulations were performed on the established model to obtain the received voltage at the receiving end in the presence of bubbles of different sizes in the silicone.

**Figure 8:**
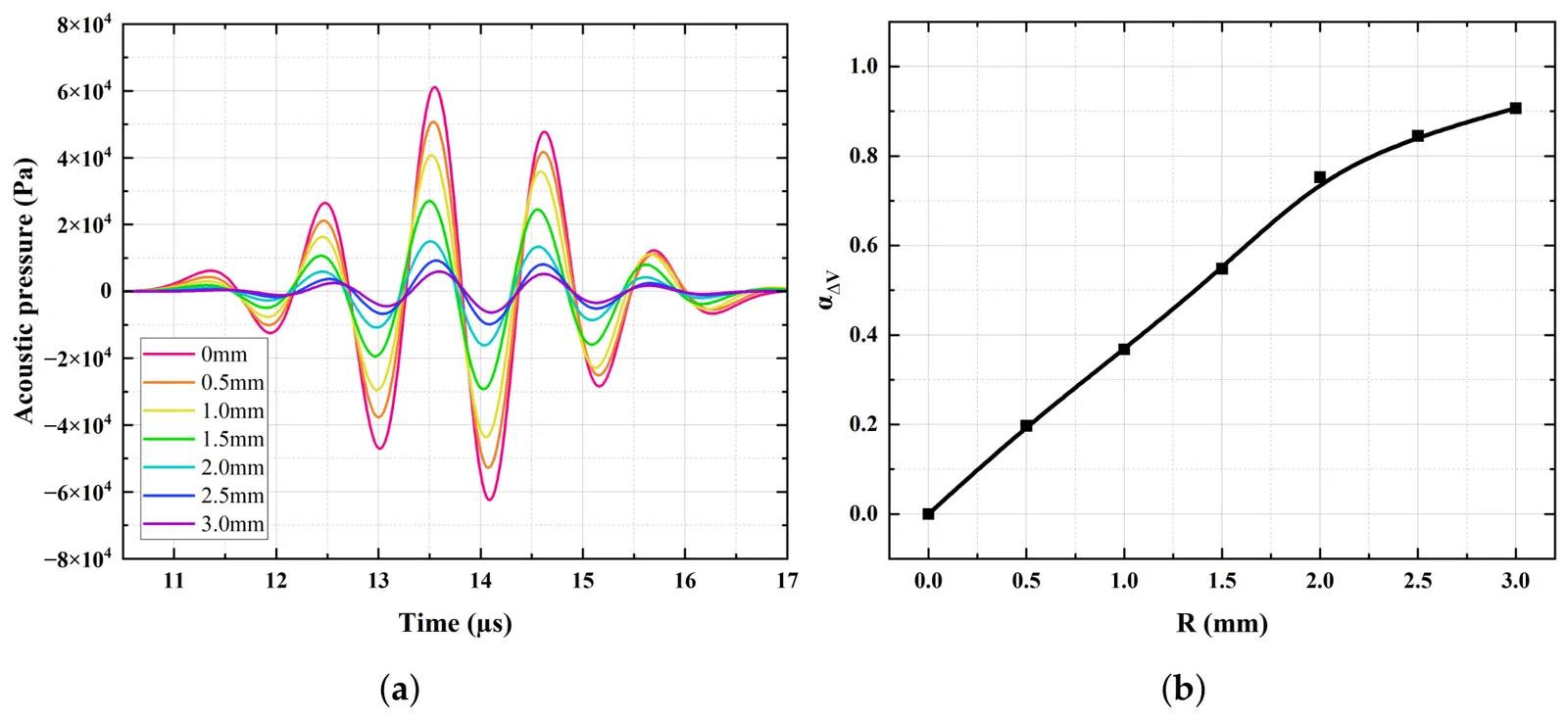
Finite element simulation analysis results. (**a**) Waveforms of received signals for bubbles with radii varying from 0 to 3 mm. (**b**) Simulated relationship between bubble size and relative change in voltage.

**Figure 9:**
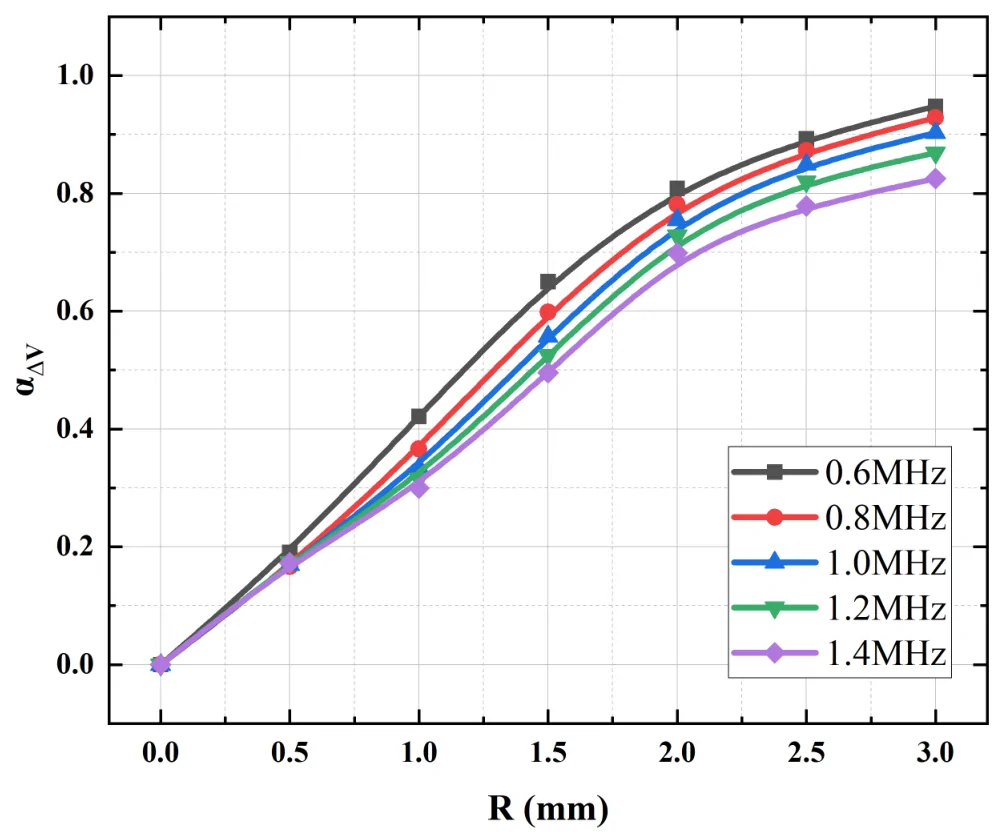
Simulated relationship between bubble size and relative change in voltage under different frequencies.

As shown in Figure 8a, these are the acoustic pressure signal plots received at the receiving end in the presence of bubbles of different sizes in silicone, and Figure 8b presents the corresponding plots of relative voltage variation. From the simulation results, it can be clearly observed that the received acoustic pressure of the wave gradually decreases with increasing bubble radius. Larger bubbles reflect more energy, resulting in greater energy attenuation. Thus, this method is feasible. Figure 9 illustrates the variation in relative voltage with bubble size under different transmitting frequencies. When the frequency increases, the acoustic attenuation of silicone itself further intensifies, thereby affecting the relative variation in acoustic pressure. An excessively low frequency leads to insufficient resolution in thickness measurement, while an excessively high frequency causes the acoustic pressure transmitted through the silicone to decrease excessively, making it difficult to obtain accurate correlation functions. In actual experimental tests, a transmitting frequency of 1 MHz will be adopted.

## 5. Detection System

The ultrasonic detection of the thickness of the measured silicone is based on the TOF. Acoustic waves propagate through the silicone and, upon encountering the silicone surface on the opposite side, generate reflections due to the significant difference in acoustic impedance. In this experiment, two CMUT arrays are placed coaxially and face-to-face on both sides of the silicone, where CMUT array No.1 is responsible for transmitting ultrasonic waves, and CMUT array No.2 is tasked with receiving the acoustic pressure signals emitted by CMUT array No.1. Meanwhile, thickness measurement is enabled based on the ultrasonic time-of-flight. Therefore, the circuit requires a pulse transmission module and an acquisition module, with precise control over both modules.

**Figure 10:**
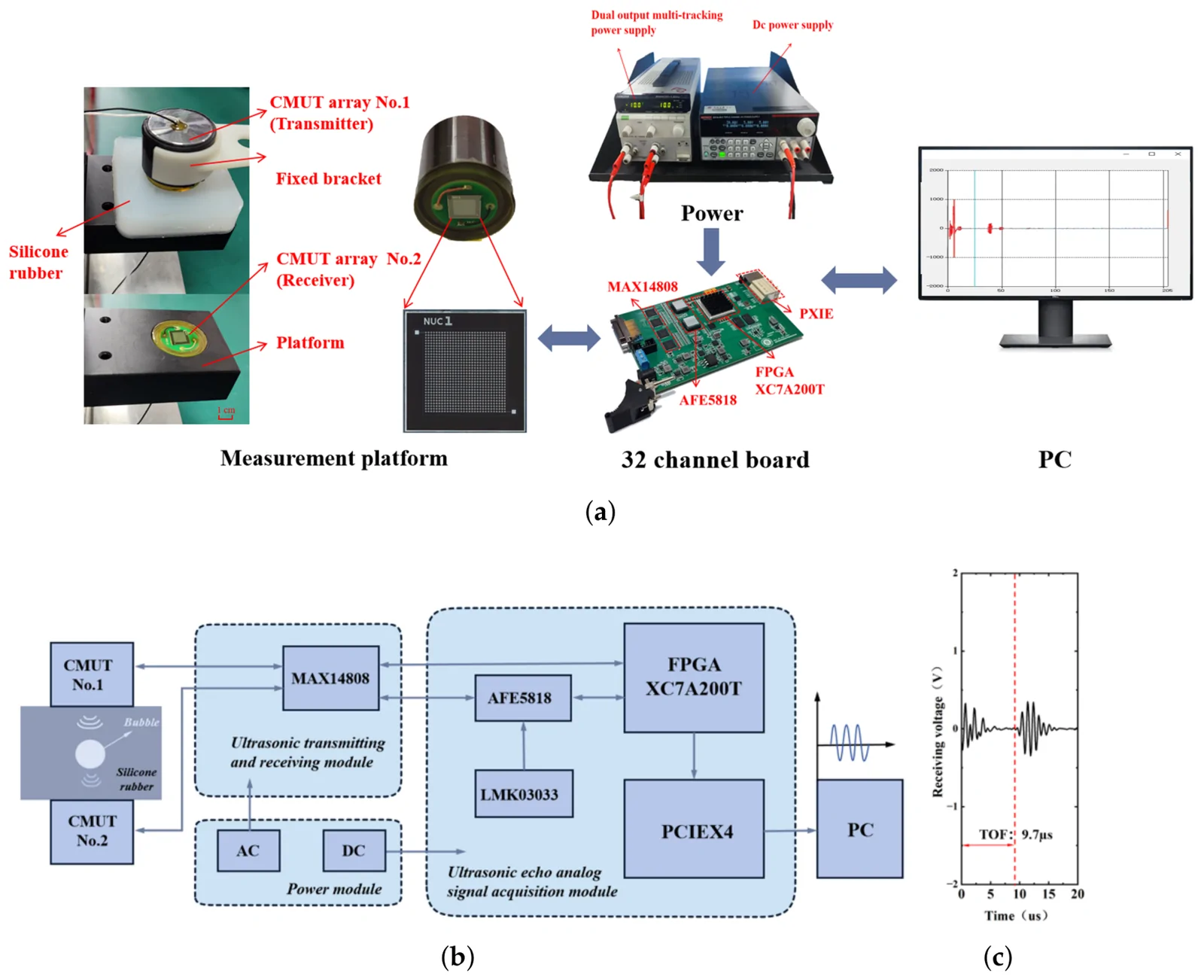
The silicone bubble monitoring system we designed and constructed. (**a**) Experimental connection diagram of the detection system. (**b**) Structural framework diagram of each module in the detection system. (**c**) Schematic diagram of ultrasonic time-of-flight.

Figure 10a and Figure 10b present the connection diagram of the detection system and the structural framework diagram of each module in the detection system, respectively. The FPGA (San Jose Xilinx, San Jose, CA, USA), MAX14808 (San Jose Maxim, San Jose, CA, USA), and AFE5818 (Dallas TI, Dallas, TX, USA) chips on the acquisition board play roles in control, signal transmission, and acquisition, respectively, and transmit data to the PC host via PXIE. The FPGA controls the MAX14808 to emit pulse signals, which excite CMUT array No.1 to transmit ultrasonic signals. The acoustic signals propagate through the silicone to CMUT array No.2, and the transmitted analog signals enter the MAX14808 connected to CMUT array No.2. The TR switch of this chip is turned on, and the signals are transmitted to the AFE5818 chip. As the core of signal conditioning, the AFE5818 integrates key modules such as a low-noise amplifier (LNA), voltage-controlled attenuator (VCAT), programmable gain amplifier (PGA), low-pass filter (LPF), and a 14/12-bit analog-to-digital converter. Among them, the LNA offers three selectable gain settings (12 dB, 18 dB, and 24 dB), and the PGA provides two gain options (24 dB and 30 dB), offering flexible support for the amplification of weak signals and adaptation to dynamic ranges, thus ensuring the accuracy and reliability of subsequent analog-to-digital conversion.

During the testing process, CMUT array No.2 was embedded in the test platform. The silicone under test was marked with precise position markers and placed on the test platform. Subsequently, the bracket holding CMUT array No.1 was adjusted to ensure that the two CMUT arrays were positioned coaxially and face-to-face on both sides of the silicone. As shown in the figure, a DC power supply (2231A-30-3, Keithley Instruments, Cleveland, OH, USA) provided a 10 V DC bias to CMUT array No.1. A dual-output multi-tracking power supply (PMM35-1.2DU, Kikusui, Yokohama, Japan) was connected to the board, supplying ±10 V to the MAX14808 chip, which accordingly generates an AC excitation amplitude of ±10 V to drive the ultrasound transmission of CMUT No.1. During operation, the MAX chip emitted three signal waves with a frequency of 1 MHz to excite CMUT array No.1. In this work, TOF and voltage amplitude of the received voltage signals were recorded. As shown in Figure 10c, when testing a silicone sample with a thickness of 10 mm, the measured ultrasonic TOF was 9.7 µs. According to the Formula (16), the ultrasonic velocity was calculated to be 1030.92 m/s, which is consistent with the theoretical value. The silicone thickness was determined by calculating it from the ultrasonic TOF obtained in each test.

## 6. Results and Discussion

**Figure 11:**
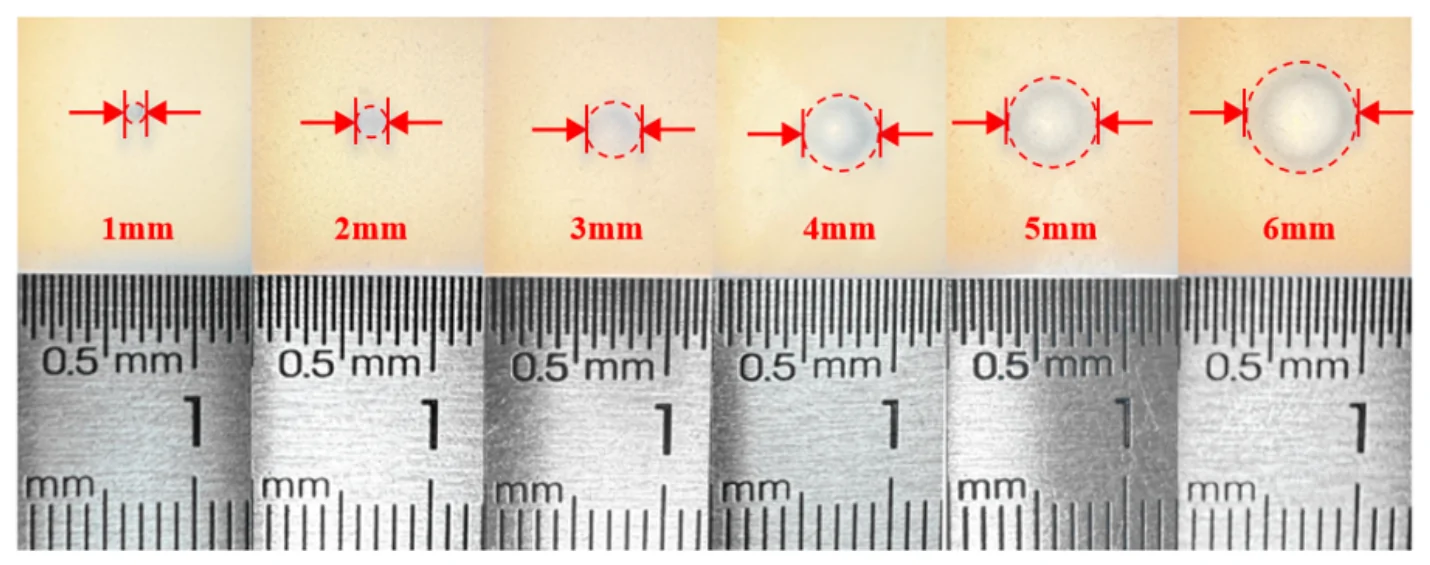
Images of bubbles with radii ranging from 0 mm to 3 mm in silicone.

**Figure 12:**
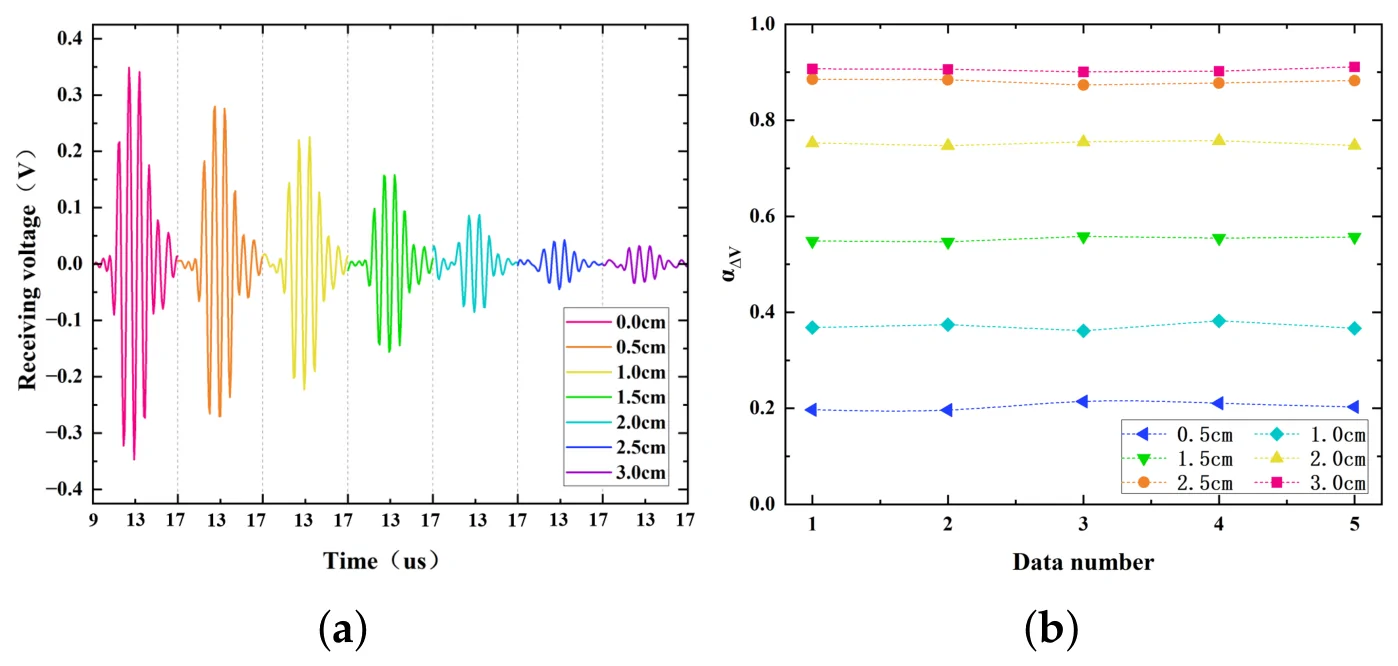
Experimental results at the same bubble position and silicone thickness. (**a**) Waveforms of signals received by CMUT array No.2 under different bubble sizes. (**b**) Repeated experimental results chart.

To validate the feasibility of using the CMUT array for bubble measurement in silicone, we fabricated silicone test blocks with the same thickness, identical bubble positions, and varying bubble sizes, as shown in Figure 11. The bubble radii ranged from 0.5 mm to 3.0 mm, adjusted in increments of 0.5 mm for testing. Positioning markers were placed on the silicone blocks to ensure that the bubbles were aligned along the axis of the two CMUT arrays. To ensure effective acoustic transmission and minimize the acoustic impedance mismatch at the interfaces, a commercial medical ultrasound coupling agent (HYNAUT, Qingdao, China) was uniformly applied between the CMUT arrays and the silicone samples. Tests were conducted with a silicone thickness of 10 mm, and the results are presented in Figure 12a. When no bubbles were present in the silicone, the voltage received by CMUT array No.2 was maximized. As the bubble size increased, the received acoustic pressure gradually decreased, demonstrating that the presence of bubbles in silicone causes acoustic wave reflection and energy attenuation. Figure 12b shows the results of multiple repeated trials. In five repeated tests, the voltage received by CMUT array No.2 exhibited slight fluctuations. The variances in the received voltage for bubble radii ranging from 0.5 mm to 3.0 mm were 0.0078 V, 0.0077 V, 0.0031 V, 0.0027 V, 0.0030 V, and 0.0022 V, respectively, indicating strong stability and repeatability of the system.

**Figure 13:**
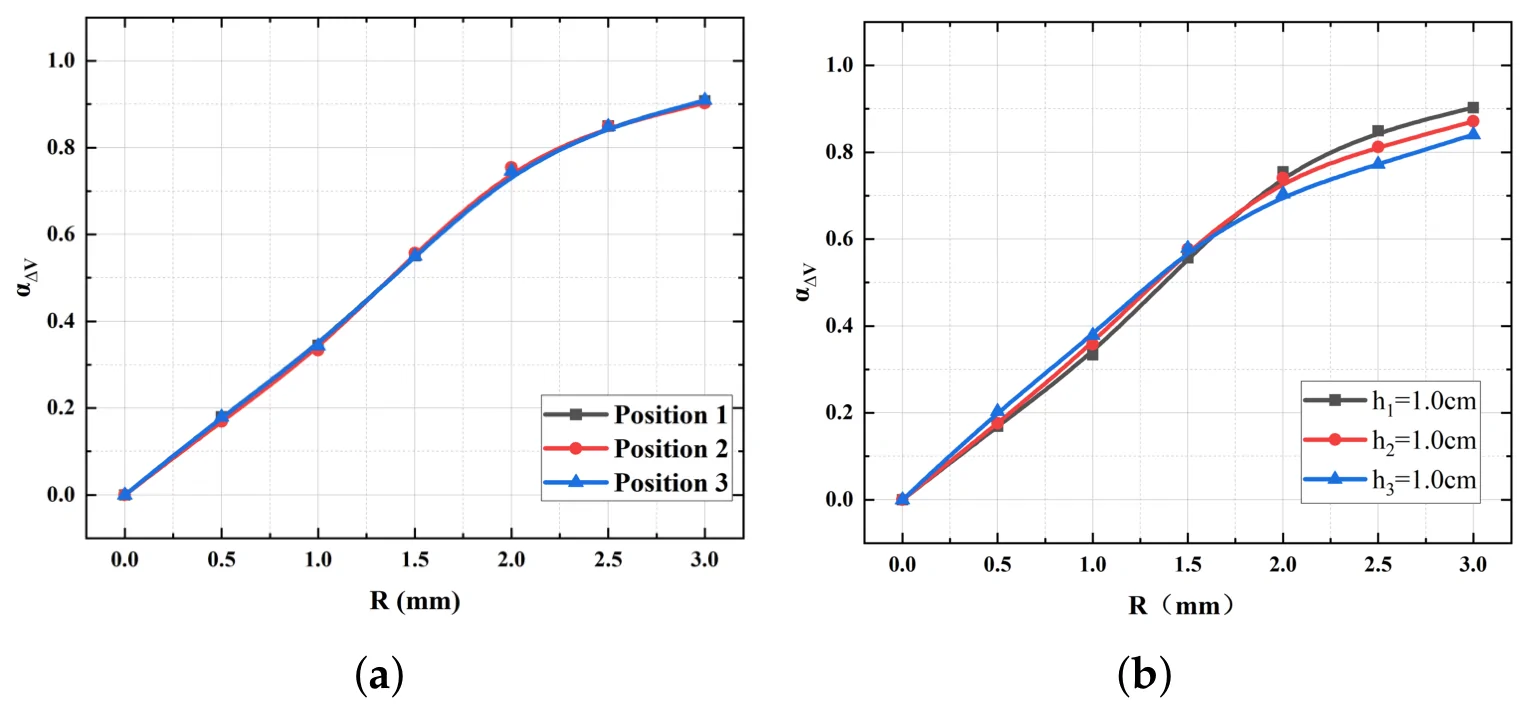
Experimental results. (**a**) Received voltage variations under bubbles with different positions. (**b**) Received voltage variations under bubbles in different thicknesses of silicone gel.

The primary purpose of this system is to inspect and identify defective products by detecting bubbles in silicone; thus, it is necessary to verify whether the position of bubbles in silicone affects the measurement of bubble size. In the aforementioned experiments, bubbles were all located at the central position of the silicone. Silicone blocks with bubbles (radii ranging from 0.5 mm to 3.0 mm) at three different positions, as shown in Figure 13a, were fabricated, and experimental tests were conducted. The test results demonstrate that the voltage variation vs. bubble radius curves for the three positions almost overlap. This indicates that the influence of the bubble position in silicone on the measurement of bubble size is negligible, as under certain conditions, acoustic energy attenuation is only related to the propagation path and bubble size.

Given that the thickness of silicone exerts a significant influence on experimental data, it is essential to conduct experimental tests to acquire relevant data. Therefore, silicone blocks with thicknesses of 10 mm, 12 mm, and 14 mm—each containing bubbles with radii ranging from 0.5 mm to 3.0 mm—were fabricated, as illustrated in Figure 13b, and experimental tests were performed. From the experimental data plot, it can be observed that the voltage variation exhibits a non-linear correlation with both bubble size and silicone thickness. Consequently, a quadratic polynomial regression model needs to be constructed.

Assuming that the voltage variation is denoted as $\alpha_{\Delta V}\left(R,H\right)$, the bubble size radius as R, and the thickness of the silicone block as H, the following prediction equation can be established:

(17) $$\alpha_{\Delta V}=\beta_{0}+\beta_{1}R+\beta_{2}H+\beta_{3}R^{2}+\beta_{4}H^{2}+\beta_{5}RH+\varepsilon$$

where $\beta_{0}$ represents the constant term (intercept); $\beta_{1}$ denotes the linear coefficient of bubble radius; $\beta_{2}$ is the linear coefficient of silicone block thickness; $\beta_{3}$ stands for the quadratic term coefficient of bubble radius; $\beta_{4}$ represents the quadratic term coefficient of silicone block thickness; $\beta_{5}$ is the coefficient of the interaction term (accounting for the combined effect of bubble radius and silicone block thickness); and ε is the random error term.

Matrices are constructed to solve the regression coefficients using the least squares method, where all experimental observations ($R_{i},H_{i},\alpha_{i}$) are formulated into the feature matrix X and the target vector C.

Solving the regression model,

(18) C=Xβ+ε

The objective function of the least squares method is to minimize the sum of squared residuals, and its analytical solution is given by

(19) $$\hat{\beta }={\left(X^{T}X\right)}^{-1}X^{T}C$$

**Figure 14:**
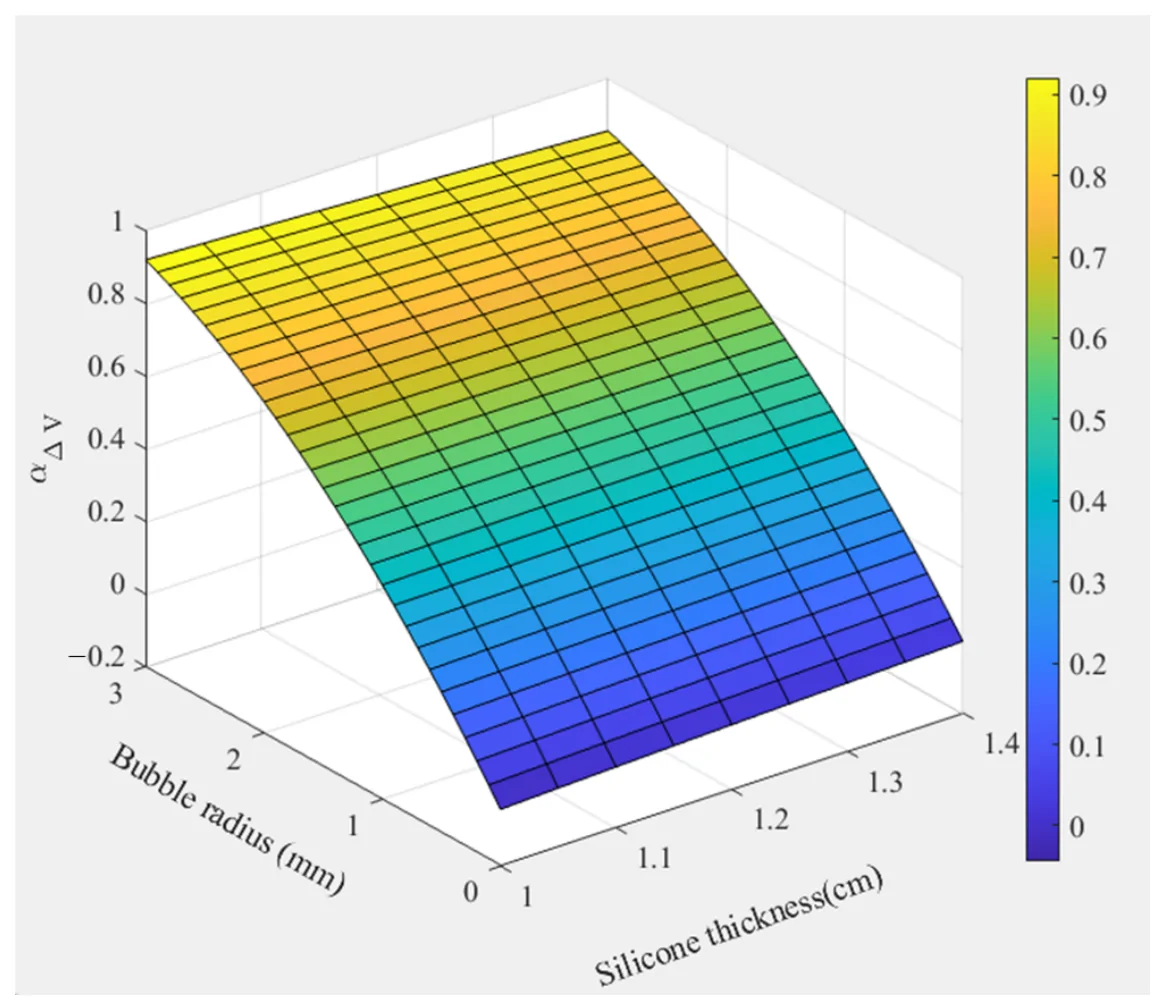
Fitted correlation surface plot.

Figure 14 presents the fitted surface of acoustic pressure variation obtained from the fitting process. By inversely determining the bubble size in silicone using this fitted surface, the detection of bubble size in silicone can be achieved.

Table 2 compares the present work with commonly used methods for bubble detection in silicone. Compared with traditional X-ray scanning methods, the bubble-testing system designed in this work is completely harmless to operators, offering a more cost-effective alternative with faster and more convenient operation. It enables one-step measurement without requiring professional expertise. The system features a rapid response time and supports real-time data acquisition. By transmitting data to the host computer, the integrated model performs calculations to directly obtain the bubble size data in silicone, which is then displayed on the host computer interface. This provides an excellent testing method for bubble detection in silicone.

## 7. Conclusions

This study presents a non-invasive ultrasonic detection system based on two CMUT arrays for identifying and sizing bubbles in industrial silicone sealing rings. By utilizing acoustic energy atten uation and analyzing variations in received voltage, the proposed system enables non-contact detection of internal bubbles without damaging the tested material. Experimental results demonstrate high measurement stability and a spatial resolution reaching 0.05 mm. Compared with conventional X-ray inspection methods, the proposed CMUT-based approach provides a safer and more cost-effective solution without ionizing radiation, while maintaining compact size and potential for real-time on-site inspection.

In this work, the experimental validation was mainly conducted using silicone samples containing a single bubble with controlled size, position, and thickness conditions, which allowed the influence of individual bubble characteristics on acoustic attenuation to be systematically investigated. However, practical silicone components may contain multiple bubbles with different sizes and spatial distributions. In such cases, interactions between multiple scattering interfaces may introduce additional acoustic attenuation and signal superposition effects, which could increase the complexity of bubble size evaluation. Therefore, further investigations involving multi-bubble samples and more advanced signal processing algorithms are required to improve the robustness of the proposed method for practical industrial applications.

Future work will focus on extending the current CMUT-based detection system toward multi-bubble characterization by combining array-scanning techniques, acoustic propagation modeling, and intelligent signal analysis methods. These improvements are expected to enhance bubble localization capability and enable more comprehensive quality control of silicone components in real manufacturing environments.

## Figures and Tables

**Table 1:** Detailed design parameters of the sensing cell in the CMUT.

Structural Parameter	Size
Device size (µm)	8800 × 8800
Number of cells	900 (30 × 30)
Vacuum chamber radius (µm)	90
Cavity height (with insulation) (µm)	0.8 (insulation 0.15)
Electrode radius (µm)	45
Film thickness (µm)	2.83
Electrode thickness (µm)	1
Silicon substrate thickness (µm)	430

**Table 2:** Performance comparison of different silicone bubble-testing systems.

Mixture	XDR-AZ350	JY-3	This Work
Technology	X-ray penetration	X-ray penetration	Ultrasonic
Harm to human body	X-ray radiation	X-ray radiation	No harm
weight	100 kg	25 kg	20 kg
Minimum size for detection	0.15 mm	0.5 mm	0.5 mm
Cost	$70,000	$70,000	$1500

## Data Availability

All data supporting this study are included in the article.
